# Assessment of Detoxification Efficacy of Irradiation on Zearalenone Mycotoxin in Various Fruit Juices by Response Surface Methodology and Elucidation of Its *in-vitro* Toxicity

**DOI:** 10.3389/fmicb.2018.02937

**Published:** 2018-11-30

**Authors:** Naveen Kumar Kalagatur, Jalarama Reddy Kamasani, Venkataramana Mudili

**Affiliations:** ^1^Toxicology and Immunology Division, DRDO-BU-Centre for Life Sciences, Bharathiar University, Coimbatore, India; ^2^Freeze Drying and Processing Technology Division, Defence Food Research Laboratory, Mysore, India

**Keywords:** mycotoxins, zearalenone, detoxification, irradiation, response surface methodology, toxicological assessment

## Abstract

Fruits are vital portion of healthy diet owed to rich source of vitamins, minerals, and dietary fibers, which are highly favorable in keeping individual fit. Unfortunately, these days, one-third of fruits were infested with fungi and their toxic metabolites called mycotoxins, which is most annoying and pose significant health risk. Therefore, there is a need to suggest appropriate mitigation strategies to overcome the mycotoxins contamination in fruits. In the present study, detoxification efficiency of irradiation on zearalenone (ZEA) mycotoxin was investigated in distilled water and fruit juices (orange, pineapple, and tomato) applying statistical program response surface methodology (RSM). The independent factors were distinct doses of irradiation and ZEA, and response factor was a percentage of ZEA reduction in content. A central composite design (CCD) consists of 13 experiments were planned applying software program Design expert with distinct doses of irradiation (up to 10 kGy) and ZEA (1–5 μg). The results revealed that independent factors had a positive significant effect on the response factor. The analysis of variance (ANOVA) was followed to fit a proper statistical model and suggested that quadratic model was appropriate. The optimized model concluded that doses of irradiation and ZEA were the determinant factors for detoxification of ZEA in fruit juices. Further, toxicological safety of irradiation mediated detoxified ZEA was assessed in the cell line model by determining the cell viability (MTT and live/dead cell assays), intracellular reactive oxygen species (ROS), mitochondrial membrane potential (MMP), nuclear damage, and caspase-3 activity. The higher level of live cells and MMP, lower extent of intracellular ROS molecules and caspase-3, and intact nuclear material were noticed in cells treated with irradiation mediated detoxified ZEA related to non-detoxified ZEA. The results confirmed that toxicity of ZEA was decreased with irradiation treatment and detoxification of ZEA by irradiation is safe. The study concluded that irradiation could be a potential post-harvest food processing technique for detoxification of ZEA mycotoxin in fruit juices. However, irradiation of fruit juices with high dose of 10 kGy has minimally altered the quality of fruit juices.

## Introduction

Fungi plays a substantial role in spoilage of agricultural commodities and produces a variety of toxic secondary metabolites called mycotoxins that are harmful to humans and farm animals (Andersen and Thrane, 2006; Van Egmond et al., 2007; Mudili et al., 2014; Venkataramana et al., 2014; Muthulakshmi et al., 2018). The fungal infestations primarily commence at pre-harvesting and post-harvesting times owed to inappropriate agronomic practices (Neme and Mohammed, 2017). The Food and Agricultural Organization (FAO) have estimated that almost one-fourth of agricultural commodities are contaminated with fungi and mycotoxins worldwide (Bryła et al., 2016). The fungi predominantly infest cereals, and its by-products (Aldred et al., 2004; Mudili et al., 2014). However, over last few decades, researchers have ascertained that fruits were as well substantially contaminated with fungi and mycotoxins and pose health at risk (Barkai-Golan and Paster, 2011; Juan et al., 2017; Škrbić et al., 2017; Zheng et al., 2017; De Berardis et al., 2018; Sandoval-Contreras et al., 2018). Mycotoxins can persevere in fruits even once the fungi have been eradicated and could diffuse into healthy portion of fruits (Taniwaki et al., 1992; Restani, 2008).

The chief mycotoxigenic fungi that infest fruits are *Aspergillus, Alternaria*, and *Penicillium* and mycotoxins produced by them are aflatoxins, ochratoxins, patulin, and alternaria (Barkai-Golan and Paster, 2011). Though, some surveys were published that *Fusarium* spp. and its mycotoxins, explicitly zearalenone (ZEA) mycotoxin is occasionally accountable for contamination of fruits (Zinedine et al., 2007). Foremost, Chakrabarti and Ghosal (1986) have reported the contamination of *F. verticillioides* and ZEA in banana fruit at pre-harvesting and post-harvesting sessions and found that contamination of ZEA was quite high (0.8–1 mg/g of fruit). Following, Blumenthal-Yonassi et al. (1988) have assessed the ZEA production by *Fusarium equiseti* strains in fruits and noticed 0.05, 3.5, 0.2, and 0.05 mg/40 g in tomato, avocado, melon, and banana, respectively. Further, Bilgrami et al. (1990) have isolated *Fusarium* species from cereals, fruits, and vegetables, and noticed that 6.8% of *Fusarium* isolates were capable to produce ZEA in the moist-rice medium under laboratory conditions. In another study, Jime and Mateo (1997) have isolated a range of *Fusarium* species, including *Fusarium graminearum* and *F. equiseti* from banana fruits and unveiled its competence to produce ZEA under laboratory conditions. The *F. graminearum* and *F. equiseti* have produced 520 and 488 μg/g, and 45 and 40 μg/g of ZEA in corn and rice cultures, respectively (Jime and Mateo, 1997). Similarly, Sharma et al. (1998) have isolated the toxigenic *F. verticillioides* from stored fruit Buchanania lanzan Spreng. (Chironji) of family Anacardiaceae native to India and observed 1–2 μg of ZEA production in broth culture. Recently, Alghuthaymi and Bahkali (2015) have assessed the toxigenic profiles of *Fusarium* species isolated from banana fruits and noticed potent producers of ZEA mycotoxin, including *F. chlamydosporum, F. circinatum, F. semitectum, F. solani, F. thapsinum*, and *F. proliferatum* and detected a maximum production of 0.912 μg/mL of ZEA in the rice culture medium under laboratory conditions. Likewise, *F. oxysporum* is one of the typical fungal contaminants of orange, pineapple, and tomato juices and could produce ZEA (Milano and López, 1991; Corbo et al., 2010; Bevilacqua et al., 2012, 2013). These scenarios have confirmed that ZEA is one of the noticeable contaminants of fruits and poses a serious threat to humans.

The ZEA is heat resilient, color, and odorless, and only know potent estrogenic mycotoxin. Many researchers have well-established the toxic effects of ZEA in cell line models and reported the involvement of caspase-3 and caspase-9-dependent mitochondrial signaling pathways in inducing the apoptotic and necrotic death of cells (Zhu et al., 2012; Venkataramana et al., 2014; Kalagatur et al., 2017). The ZEA primarily elevates the intracellular ROS and lipid peroxidation, and incites phosphorylation of histone H3, aberrations of chromosome and exchange of sister chromatid and instabilities in the mitotic index, DNA fragments and adduct formation, micronuclei development, inhibits DNA and RNA syntheses, and finally affects the cell viability (Kouadio et al., 2005; Gao et al., 2013). The International Agency for Research on Cancer (IARC) has evaluated the genotoxic and carcinogenic effects of ZEA under *in-vitro* conditions and recommended under Group 3 carcinogens (IARC, 1993). In view of the taxological effects, many nations and regulatory bodies, i.e., European Union (EU), World Health Organization (WHO), and Food and Agriculture Organization (FAO) have recommended stringent regulations and management practices to lower ZEA levels in food and feed matrices (European Commission, 2006; JECFA, 2011; Kalagatur et al., 2015).

In the contemporary concern, physical process, especially γ-radiation has attained great demand due to its prompt and robust action (Karlovsky et al., 2016; Kalagatur et al., 2018b,c). The γ-radiation is the shorter wavelength of electromagnetic radiation and offers high penetrating power of above 100 keV. The irradiation processing improves the microbiological safety and prolongs the shelf life of food without much substantially change in physical, chemical, and nutritional properties (Calado et al., 2014; Kalawate and Mehetre, 2015; Choi and Lim, 2016). Furthermore, WHO and FAO of the United Nations have specified that irradiation of some niche products and markets up to dosage rate of 25 kGy is safe and endorsed as appropriate decontamination technique in agriculture and food industry (FAO/IAEA/WHO, 1999).

Best of our knowledge, detoxification efficacy of irradiation on ZEA in fruit juices has not been reported, and this is the first attempt. In the present study, detoxification efficiency of irradiation on ZEA was established in distilled water, and fruit juice of orange, pineapple, and tomato by response surface methodology (RSM). Furthermore, toxicological safety of irradiation mediated detoxified ZEA was assessed in the cell line model by determining cell viability (MTT and live/dead cell assays), intracellular ROS, MMP, nuclear damage, and caspase-3 activity.

## Materials and methods

### Chemicals and reagents

Standard ZEA (HPLC grade, 99% pure), caspase-3 assay kit, rhodamine 123, 4′,6-diamidino-2-phenylindole (DAPI), dichloro-dihydro-fluorescein diacetate (DCFH-DA), and [3-(4,5-dimethylthiazol-2-yl)-2,5-diphenyltetrazolium bromide] (MTT) were received from Sigma-Aldrich (Bengaluru, India). The live/dead cell assay kit was from Invitrogen Molecular Probes (Bengaluru, India). The Dulbecco's phosphate-buffered saline pH 7.4 (DPBS), antibiotic solution (streptomycin and penicillin), fetal bovine serum (FBS), Dulbecco's modified Eagle's medium (DMEM), and plasticware were obtained from HiMedia (Mumbai, India). Acetonitrile, methanol, dimethyl sulfoxide (DMSO), distilled water, and other chemicals of superior grade were bought from Merck Millipore Corporation (Bengaluru, India).

### Preparation of fruit juices

A fresh orange, pineapple, and tomato were obtained from the regional agricultural market of Mysuru, Karnataka state, India, and washed rigorously with distilled water. The endocarp of orange, fine pieces of pineapple and tomato were squeezed and attained the juice. Further, debris was separated from juice by filtering through 0.45 μm syringe filter and clear juice were used in detoxification studies.

### Detoxification of ZEA by irradiation

#### Design of experiment

The detoxification efficiency of irradiation on ZEA was assessed in distilled water and clear fruit juice of orange, pineapple, and tomato accomplishing the statistical program RSM. A central composite design (CCD) consists of 13 experiments were planned with distinctive doses of irradiation (up to 10 kGy) and ZEA (1 to 5 μg) applying software program Design-Expert trial version 10 (State–Ease, Minnesota, USA) (Atkinson and Donev, 1992; Whitcomb and Anderson, 2004; Anderson and Whitcomb, 2016; Kalagatur et al., 2018a). The type, unit, range, coded levels, mean, and standard deviation of independent variables are shown in Supplementary Table 1. The response factor was a percentage of ZEA reduction in distilled water and fruit juices after exposing to irradiation. The optimized design intended for the study was generated by polynomial regression analysis.

#### Irradiation process

The stock solution of ZEA (1 mg/mL) was prepared in acetonitrile and further different test concentrations of ZEA was made in 1 mL of distilled water and clear fruit juice of orange, pineapple, and tomato (1–5 μg/mL) following CCD as shown in Table 1 and subjected to irradiation. Cobalt 60 was a source of γ-rays and irradiation was carried out at 35°C with a dosage rate of 5.57 kGy per hour under Gamma irradiation chamber-5000. The Ceric-cerous standard dosimeter that fixed on top and surface bottom of the sample was used to measure the absorbed dose of γ-radiation. The uniformity of irradiation dose (D_max_/D_min_) was maintained at 1.01 (Reddy et al., 2015).

**Table 1:** Central composite design (CCD) for evaluation of detoxification efficiency of irradiation on zearalenone (ZEA) in distilled water and fruit juice of orange, pineapple, and tomato.

**Run order**	**Independent factors**		**Response factor (percentage of ZEA reduction)**			
	**A: Conc. of ZEA (μg)**	**B: Dose of irradiation (kGy)**	**Distilled water**	**Orange juice**	**Pineapple juice**	**Tomato juice**
1	4.41 (+1)	1.46 (−1)	11.73 ± 0.22^a^	10.99 ± 0.40^a^	10.05 ± 0.32^a^	11.46 ± 0.26^a^
2	1.58 (−1)	8.53 (+1)	83.71 ± 2.09^b^	82.63 ± 1.17^b^	81.97 ± 1.89^b^	81.59 ± 1.93^b^
3	4.41 (1)	8.53 (+1)	52.09 ± 1.36^c^	50.42 ± 0.91^c^	51.71 ± 0.94^c^	52.81 ± 0.80^c^
4	3.00 (0)	5.00 (0)	46.92 ± 0.85^d^	44.35 ± 0.97^d^	43.60 ± 0.66^d^	45.08 ± 0.88^d^
5	1.58 (−1)	1.46 (-1)	27.46 ± 0.71^e^	26.88 ± 0.39^e^	25.92 ± 0.41^e^	26.37 ± 0.56^e^
6	5.00 (+α)	5.00 (0)	34.07 ± 0.78^f^	33.29 ± 0.64^f^	32.16 ± 0.83^f^	33.64 ± 0.80^f^
7	3.00 (0)	5.00 (0)	47.02 ± 0.92^dg^	46.09 ± 0.70^dg^	45.39 ± 0.82 ^dg^	45.11 ± 0.67 ^dg^
8	3.00 (0)	10.00 (+α)	71.05 ± 0.81^h^	69.47 ± 1.14^h^	70.54 ± 0.89^h^	70.53 ± 1.45^h^
9	3.00 (0)	5.00 (0)	41.89 ± 0.69^dgi^	41.70 ± 0.47^dgi^	40.83 ± 0.63^dgi^	40.12 ± 0.81^dgi^
10	3.00 (0)	5.00 (0)	43.55 ± 0.77^dgij^	43.38 ± 0.41^dgij^	42.62 ± 0.65^dgij^	42.07 ± 0.82^dgij^
11	1.00 (–α)	5.00 (0)	72.51 ± 0.84^k^	70.02 ± 1.01^k^	68.37 ± 1.77^k^	69.19 ± 0.94^k^
12	3.00 (0)	5.00 (0)	43.11 ± 0.29^dgijl^	41.20 ± 0.50^dgijl^	40.59 ± 0.43^dgijl^	42.30 ± 0.61^dgijl^
13	3.00 (0)	0.00 (–α)	0.00^m^	0.00^m^	0.00^m^	0.00^m^

*The statistical analysis was executed by Tukey's multiple comparison test and the columns with different alphabetic letters were statistically significant (p < 0.05) in respective study*.

#### Quantification of ZEA by HPLC

Following irradiation treatments, quantification of ZEA was carried out using HPLC system (Shimadzu, Kyoto, Japan) as per methodology of Kumar et al. (2016) and HPLC conditions are provided in Supplementary Table 2. The quantification of ZEA was deducted from the calibrated curve of standard ZEA. For constructing calibration curve, different dilutions of ZEA were made in water (100 ng−1 μg/mL) from stock solution of ZEA (1 mg/mL in acetonitrile) and 25 μL was injection into HPLC. The calibration curve was constructed with area of peak vs concentration of ZEA. The precise of the calibration curve was judged by linear regression analysis. The attained regression curve has shown decent linearity with a coefficient of determination (*R*^2^) of 0.9932. The limit of detection (LOD) was the signal-noise ratio of 3 and limit of quantification (LOQ) was the signal-noise ratio of 10. The LOD and LOQ were noticed as 22 and 86 ng/mL, respectively. The percentage of recovery of technique was 96.58 for 1 μg/mL of ZEA. The accuracy of the technique for inter-day was expressed by Relative Standard Deviation (RSD%) and it was 7.31%.

The percentage of ZEA reduction (response factor) in irradiated test samples was deduced from the formula,

$$\text{ZEA reduction }(\%)\;=\frac{{\text{Z}}_{\text{PI}}-{\text{Z}}_{\text{AI}}}{{\text{Z}}_{\text{PI}}}\times 100$$

Where, Z_PI_ was a concentration of ZEA prior irradiation and Z_AI_ was a concentration of ZEA after irradiation.

#### Optimization of design

The regression analysis of the response factor (percentage of ZEA reduction) was assessed by the second-order polynomial equation. The design was optimized by considering variables of polynomial regression at *p* < 0.05. Furthermore, precision of the optimized model was approved by asserting the coefficient of determination (*R*^2^). In conclusion, accuracy of the optimized design was assessed by normal plot residuals, Box-Cox, actual vs. predicted, and 3-D response plots (Anderson and Whitcomb, 2016). The second-order polynomial equation applied for the analysis of variables as follows,

$$\begin{array}{l} \text{Y}={\text{β}}_{0}+\overset{\text{n}}{\underset{\text{i}=\text{1}}{\sum }}\beta_{\text{i}}x_{\text{i }}+\;\overset{\text{n}}{\underset{\text{i}=\text{1}}{\sum }}\beta_{\text{ii}}{\text{x}}_{\text{i}}^{\text{2}}+\overset{\text{n}}{\underset{\text{i}\neq \text{j}=\text{1}}{\sum }}\beta_{\text{ii}}{\text{x}}_{\text{i}}{\text{x}}_{\text{ij}} \end{array}$$

Where, “0” represents suitable response value at center point of the model. The linear, quadratic, cross-product terms of the model were symbolized by *i, ii*, and *ij*, respectively. The total number of independent variables in the model were symbolized by alphabetical letter “n.”

### *In-vitro* toxicological examination of detoxified ZEA

The conclusive aim for the study was to assess the toxicological safety of irradiation mediated detoxified ZEA. The toxic effects of irradiation mediated detoxified ZEA was appraised by comparing with non-detoxified ZEA in *in-vitro* cell line model by determination of cell viability (MTT and live/dead cell), intracellular ROS, MMP, nuclear damage, and caspase-3 activity.

#### Cell culture and maintenance

The macrophage cell line (RAW 264.7) of *Mus musculus* was obtained from the National Center for Cell Science, India (NCCS). The cells were maintained in moisturized incubator at 5% CO_2_ and 37°C. The growth media for cell line was DMEM completed with 10% FBS, 50 mU/mL of penicillin, and 50 μg/mL of streptomycin. The cells were grown-up in 75 cm^2^ flasks and confluent cells have employed in the further experiments.

#### Experimental design

In the present study, test samples of ZEA (3 μg/mL prepared in distilled water) were distinctly subjected to detoxification with 5 and 10 kGy of irradiation. The test sample not treated with irradiation was considered as non-detoxified ZEA. Following, test samples were dried out by lyophilization and suspended in 100 μL of DMEM devoid of FBS and used for *in-vitro* toxicological analysis. The exposure of test samples to cells was categorized into following groups. Group A: Cells were treated alone with 100 μL of DMEM devoid of FBS (control). Group B: Cells were treated with non-detoxified ZEA (3 μg) in 100 μL of DMEM devoid of FBS. Group C: Cells were treated with detoxified ZEA (3 μg) of 5 kGy irradiated in 100 μL of DMEM devoid of FBS. Group D: Cells were treated with detoxified ZEA (3 μg) of 10 kGy irradiated in 100 μL of DMEM devoid of FBS.

#### Cell culture treatment

Approximately, 5 × 10^3^ cells were seeded in 96-well cell culture plates and allowed to adhere for 12 h. The cells were treated with different experimental groups as aforementioned in “experimental design” and incubated for 12 h. The volume of the media in all experimental groups was maintained as 100 μL/well. Following, plates were separately employed for various toxicological assessments, i.e., cell viability (MTT and live/dead cell), intracellular ROS, MMP, nuclear staining, and caspase-3 assays.

##### MTT assay

Following, treatments and incubation as detailed in section “Cell culture treatment.” The cells were washed for twice with DPBS and treated with 100 μL of MTT reagent (5 mg/mL in DPBS) for 4 h (Venkataramana et al., 2014). Following, MTT solution was replaced with 100 μL of DMSO to liquefy the formazan crystals for 30 min and optical density was measured at 570 nm using a multiplate reader (Synergy H1, BioTek, USA). The cell viability was determined in percentage with respect to control sample (100%).

##### Live/dead cell assay

Following, treatments and incubation as detailed in section “Cell culture treatment.” The cells were washed with DPBS for two times and stained with dyes (2 μM of calcein AM and 4 μM of ethidium homodimer-1) of live/dead cell assay kit as per directions from the manufacturer (Haugland et al., 1994). Subsequently, cells were washed with DPBS and fluorescence images were captured under green fluorescent protein (GFP) and red fluorescent protein (RFP) filters using an inverted fluorescence microscope (EVOS, Life Technologies, USA). The optical density was measured at excitation and emission of 485 and 530 nm for calcein AM, and 530 nm and 645 nm for ethidium homodimer-1, respectively using a multimode plate reader (Synergy H1, BioTek, USA) and the percentage of live and dead cells were calculated as per methodology of Garcia-Recio et al. (2015). The results were expressed with respect to control sample (100%).

##### Analysis of intracellular ROS molecules

Following, treatments and incubation as detailed in section “Cell culture treatment.” The cells were washed with DPBS for twice and stained with 5 μM of DCFH-DA for 5 min. Subsequently, cells were subjected to DPBS wash and optical density was measured at excitation of 495 nm and emission of 550 nm using a multiplate reader (Synergy H1, BioTek, USA). The fluorescent images were captured under GFP filter using an inverted fluorescence microscope (EVOS, Life Technologies, USA). The results were expressed as a percentage of intracellular ROS release with respect to the control (Venkataramana et al., 2014).

##### Analysis of mitochondrial membrane potential (MMP)

Following, treatments and incubation as detailed in section “Cell culture treatment.” The cells were washed with DPBS for twice and stained with rhodamine 123 (5 μM) in DPBS for 15 min and again washed with DPBS. The fluorescent images were captured under GFP filter using an inverted fluorescence microscope (EVOS, Life Technologies, USA). Also, optical density was measured at excitation and emission of 511 and 534 nm, respectively using a multiplate reader (Synergy H1, BioTek, USA) and results of test samples were expressed with respect to the control (Venkataramana et al., 2014).

##### Analysis of nuclear damage

Following, treatments and incubation as detailed in section “Cell culture treatment.” The cells were subjected to wash for twice with DPBS and stained with 5 μM of DAPI for 15 min. Next, cells were again washed with DPBS and fluorescent images were captured under DAPI filter using an inverted fluorescence microscope (EVOS, Life Technologies, USA).

##### Analysis of caspase-3 activity

Following, treatments and incubation as detailed in section “Cell culture treatment.” The cells were washed with DPBS for twice and exposed to reagents of caspase-3 kit and optical density was recorded at an excitation of 360 nm and emission of 460 nm using a multiplate reader (Synergy H1, BioTek, USA) following the directions from the manufacturer (Riss et al., 2016). The quantification of caspase-3 activity was determined from a standard of the fluorescent molecule 7-amino-4-methyl coumarin (AMC) release as per instructions of kit. The results were expressed in percentage of caspase-3 release with respect to the control (Lozano et al., 2009).

### Quality assessment of fruit juices treated with irradiation

A quantity of 10 mL fresh juice of orange, pineapple, and tomato were treated with different doses of irradiation, i.e., 2.5, 5, 7.5, and 10 kGy. The juice sample not treated with irradiation was referred as control. Following, quality of fruit juices of control and test samples were evaluated by sensory (appearance, aroma, consistency, and taste), pH, acidity, total soluble solids, total phenolic and flavonoid content, and total antioxidant activity.

#### Sensory evaluation

The sensory evaluation was carried out by 13 semi-trained panelists on the 9-point hedonic scale (1: Extremely poor. 2: Very poor. 3: Poor. 4: Fair above poor. 5: Fair. 6: Good above fair. 7: Good. 8: Very good. 9: Excellent) as per Murray et al. (2001). Further, over-all acceptability of fruit juices was also carried out by 13 semi-trained panelists on 9-point hedonic scale (1: Dislike extremely. 2: Dislike very much. 3: Dislike moderately. 4: Dislike slightly. 5: Neither like nor dislike. 6: Like slightly. 7: Like moderately. 8: Like very much. 9: Like extremely) as per Murray et al. (2001).

#### Determination of acidity and pH

The pH of the samples was determined using an Orion Expandable Ion Analyzer EA 940 pH meter (Expotech, USA). The total titratable acidity of the samples was measured following official methods of analysis of AOAC International 1996 and expressed as % citric acid. The total soluble solids in terms of °Brix was determined using a Carl Zeiss 844976 Jena refractometer as per official methods of analysis of AOAC International 1996.

#### Estimation of total phenolic content

The total phenolic content of fruit juice was estimated by Folin-Ciocalteau assay. Briefly, 0.5 mL of fruit juice was diluted with distilled water by three times and blended with 0.5 mL of 7.5% sodium carbonate solution and 0.25 mL of Folin-Ciocalteau reagent. The obtained mixture was incubated at 27 ± 2°C for 30 min in the dark and absorbance was recorded at 765 nm using multimode plate reader (Synergy H1, BioTek, USA). Gallic acid was used as the reference and obtained results was stated as mg of gallic acid equivalents per mL (mg GAE/mL).

#### Estimation of total flavonoid content

The total flavonoid content in fruit juice was determined by aluminum chloride colorimetric method. Briefly, 0.5 mL of juice was added to 70 μL of sodium nitrite solution (5%) and incubated for 5 min at 27 ± 2°C. Subsequently, mixture was blended with 0.5 mL of sodium hydroxide (1 M), 0.15 mL of aluminum chloride (10%), and 1.3 mL of deionized water and incubated for 5 min at 27 ± 2°C. Following, absorbance was measured at 415 nm using a multimode plate reader (Synergy H1, BioTek, USA). Catechin was used as reference and results were expressed as mg of catechin equivalents per mL (mg CE/mL).

#### Determination of total antioxidant activity

The total antioxidant activity of fruit juice was determined by DPPH radical scavenging assay. Briefly, 100 μL of fruit juice was blended with 3 mL of 4% DPPH methanolic solution. The mixture was incubated at 27 ± 2°C for 20 min in the dark and absorbance was measured at 517 nm using multimode plate reader (Synergy H1, BioTek, USA). The DPPH methanolic solution not blended with fruit juice was conceded as blank. The total antioxidant activity of the test sample was calculated using following formula,

$$\text{DPPH }(\%\text{ inhibition})=\frac{\left({\text{Ab}}_{\text{b}}-{\text{Ab}}_{\text{t}}\right)}{{\text{Ab}}_{\text{b}}}\times 100$$

Where, Ab_b_ and Ab_t_ were absorbance of blank and test samples, respectively.

### Statistical analysis

The experiments were set up independently for six times, and results were expressed as mean ± standard deviation. The CCD and actual and predicted analysis of RSM, and *in-vitro* toxicological data were analyzed by one-way ANOVA following the Tukey's multiple comparison test using GraphPad Prism trial version 7 software application and value of *p* < 0.05 was considered statistically significant. Though, quality assessment of irradiated fruit juices was compared with control by Dunnett's test using GraphPad Prism trial version 7 software application and *p* < 0.05 was considered as statistically significant.

## Results and discussion

### Detoxification of ZEA by irradiation

Knowledge on the detoxification efficiency of irradiation for mycotoxins is insufficient, and most of the studies in the literature were addressed on aflatoxins (Calado et al., 2014). Till a date, no study was focused upon the application of irradiation for detoxification of standard ZEA (HPLC grade, 99% pure) in liquid food matrices, and this is the first report. Though, (Hooshmand and Klopfenstein, 1995) and (Aziz et al., 1997) have reported the detoxification action of irradiation on ZEA in solid food matrices (maize, wheat, and soybean). In these studies, detoxification competence of irradiation on ZEA was unclear and toxic effects of detoxified ZEA was not assessed. Henceforth, present study was focused on to establish detoxification efficiency of irradiation on standard ZEA in distilled water and fruit juice of orange, pineapple, and tomato by RSM statistical program. Also, toxic effects of detoxified ZEA was assessed under *in-vitro* studies by comparing with non-detoxified ZEA.

In the present study, RSM method was applied to assess the interface among the two independent variables (ZEA and γ-radiation) on the percentage of ZEA reduction (response factor) in distilled water and fruit juices. The design with variables (different dosage of ZEA and γ-radiation) and actual responses (% of ZEA reduction) is shown in Table 1. The attained CCD results were analyzed by second order polynomial equation to fit appropriate response surface design.

The analysis of variance (ANOVA) was designated to fit suitable statistical model between independent variables and response factor, and to assess the model statistics for the optimization process. A quadratic model was highly applicable for all the responses and ANOVA results are presented in Supplementary Tables 3–6. All attained models were presented larger *F*-value and smaller *p*-value. On the other hand, lack of fit of attained designs was not significant. The goodness of the designs was estimated from the coefficient of determination (*R*^2^). The obtained *R*^2^-value of 0.9953 (distilled water), 0.9969 (orange juice), 0.9969 (pineapple juice), and 0.9960 (tomato juice) concluded that 99.53, 99.69, 99.69, and 99.60% of variations in the study possibly will be explained by design models of distilled water, orange, pineapple, and tomato fruit juices, respectively (Supplementary Table 7). Likewise, predictable *R*^2^-value was much closer to the adjusted *R*^2^-value in all the responses, and attained differences were quite in agreement (Supplementary Table 7). Moreover, adequate precision was higher than 4.0 in all responses and which concluded that attained design has an adequate signal and comfortable to navigate in the design space. The coefficient of independent variables in terms of coded factors for second order regression equation for responses was obtained as,

$$\begin{array}{l} \text{Percentage of ZEA reduction in distilled water }\\ \;=\;+\;44.50\;-\;12.71\;*\text{A }+\;24.64\;*\text{B }-\;3.97\;*\text{A }*\text{B }\\ \;+\;4.23\;*\text{A}2\;-\;4.65\;*\text{B}2\\ \text{Percentage of ZEA reduction in orange juice }\\ \;=\;+\;43.34\;-\;12.51\;*\text{A }+\;24.18\;*\text{B }-\;4.08\;*\text{A }*\text{B }\\ \;+\;4.04\;*\text{A}2\;-\;4.42\;*\text{B}2\\ \text{Percentage of ZEA reduction in pineapple juice }\\ \;=\;+\;42.61\;-\;12.17\;*\text{A }+\;24.68\;*\text{B }-3.60\;*\text{A }*\text{B }\\ \;+\;3.74\;*\text{A}2\;-\;3.76\;*\text{B}2\\ \text{Percentage of ZEA reduction in tomato juice }\\ \;=\;+\;42.94\;-\;11.75\;*\text{A }+\;24.54\;*\text{B }-\;3.47\;*\text{A }*\text{B }\\ \;+\;4.17\;*\text{A}2\;-\;3.91\;*\text{B}2 \end{array}$$

**Figure 1:**
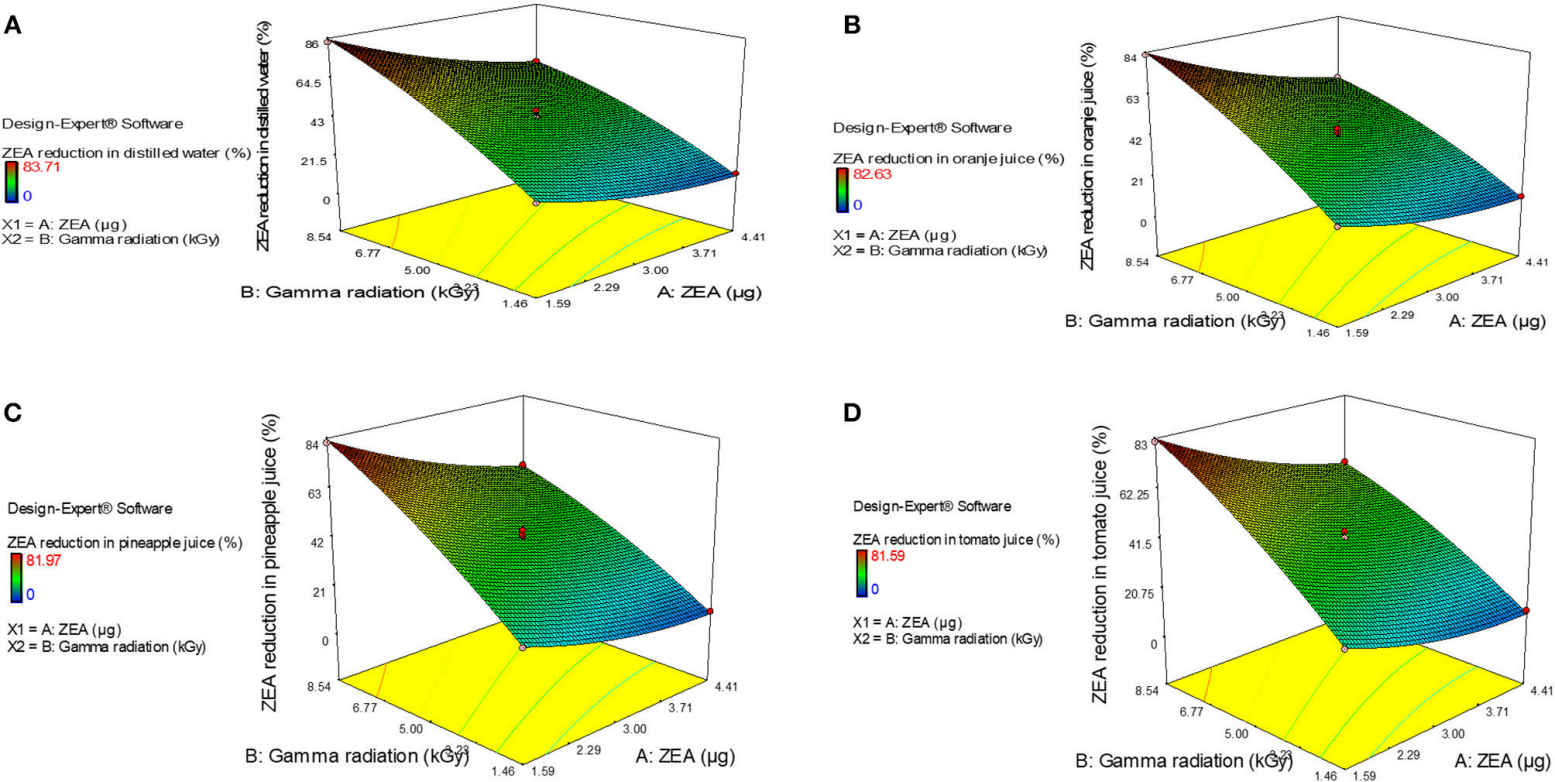
3-D response plots for interactive effect of variations in independent variables, i.e., zearalenone (ZEA) and irradiation on response factor (percentage of ZEA reduction) in **(A)** distilled water, **(B)** orange juice, **(C)** pineapple juice, and **(D)** tomato juice.

Furthermore, normal plot residuals, Box-Cox, and actual vs. predicted plots were considered to evaluate the accuracy of optimized design. The external studentized residuals were closely distributed and followed the normal plot residuals (Supplementary Figure 1), which showed that residuals were in linear behavior and the attained design was accurate (Anderson and Whitcomb, 2016). The Box-Cox plots of responses were considered to determine the most appropriate power law transformation. In the obtained design, best recommend transform (λ) were noticed for all responses (Supplementary Figure 2). The obtained λ-value was close to the current value of 1 for none and, which indicated that responses were followed Box-Cox power transform and attained design was accurate. In Supplementary Figure 3, obtained data points of actual were close to predicted and generated decent *R*^2^-value for all responses. Finally, fitted second-order polynomial equation was expressed in 3D-surface plots in Figure 1 to represent the interactive effect of variations in independent variables on responses. These figures have revealed that levels of ZEA have more impression trailed by altered doses of irradiation. Thus, diagnostic plots were concluded that optimized design well-appropriate and significant. Finally, the predicted values of the design were verified with actual values of optimized design to conclude the appropriateness of the design. The actual values of the experiment were in agreement with predicted values of the study (Table 2).

**Table 2:** Assessment of proposed predicted values of design with actual values of study.

**S. No**	**Independent factors**		**Responses (percentage of ZEA reduction in distilled water, and fruit juice of orange, pineapple, and tomato)**							
			**Predicted**				**Actual**			
	**A: ZEA (μg)**	**B: Irradiation (kGy)**	**Distilled water**	**Orange juice**	**Pineapple juice**	**Tomato juice**	**Distilled water**	**Orange juice**	**Pineapple juice**	**Tomato juice**
1	1.6	6.0	69.12 ± 1.11	67.49 ± 0.88	66.19 ± 0.89	66.43 ± 1.01	68.58 ± 1.72^a^	66.09 ± 1.80^a^	68.16 ± 0.93^a^	68.07 ± 0.31^a^
2	2	6.1	63.71 ± 0.95	62.22 ± 0.75	61.21 ± 0.76	61.36 ± 0.86	62.07 ± 0.81^b^	61.44 ± 0.66^b^	63.18 ± 0.81^b^	60.32 ± 1.16^b^
3	3	6.3	52.91 ± 0.91	51.62 ± 0.72	51.16 ± 0.74	51.41 ± 0.83	51.02 ± 0.79^c^	52.72 ± 1.02^c^	50.90 ± 0.93^c^	49.67 ± 0.82^c^
4	1.6	8.5	84.95 ± 1.63	83.28 ± 1.28	82.59 ± 1.31	82.51 ± 1.47	87.39 ± 1.04^d^	82.41 ± 0.79^d^	83.80 ± 1.07^d^	79.30 ± 0.93^d^
5	2	8.5	78.21 ± 1.33	76.66 ± 1.03	76.35 ± 1.07	76.21 ± 1.20	75.08 ± 1.66^e^	72.29 ± 1.19^e^	74.48 ± 1.03^e^	72.16 ± 1.02^e^
6	3	8.5	64.32 ± 1.07	62.94 ± 0.85	63.36 ± 0.86	63.40 ± 0.97	67.54 ± 0.92^af^	64.10 ± 1.32^af^	61.19 ± 0.77^bf^	60.08 ± 1.28^bf^

*The statistical analysis was executed by Tukey's multiple comparison test and the columns with different alphabetic letters were statistically significant (p < 0.05) in respective study*.

As we have noticed, ZEA in an aqueous solution can be effectively detoxified by irradiation and it is mostly mediated by the reactive species that are produced from radiolysis of water. The radiolysis of water by irradiation is a quick process, which takes only about 10^−6^ s and generates positive-charged water radicals (H_2_O^+^) and negative-charged free electrons (e^−^). Furthermore, series of cross-combination and recombination reactions between H_2_O^+^ and e^−^ leads to the formation of highly reactive species, i.e., ${\text{e}}_{\text{aq}}^{-}$, H^∙^, H_2_, HO^∙^, OH^−^, ${\text{HO}}_{2}^{∙}$, H_3_O^+^, and H_2_O_2_ (Le Caër, 2011). These highly reactive molecules formed as a result of radiolysis of water could attack and cleave the hydrogen, methyl, and hydroxyl molecules of ZEA and thus degrade the ZEA (Shier et al., 2001). The present study has proven the detoxification efficiency of the irradiation process on ZEA in aqueous solution of water, and fruit juice of orange, pineapple, and tomato. However, further research is needed on extraction, purification, and structural elucidation of radiolytic products of ZEA to reveal the precise process of ZEA detoxification.

### *In-vitro* toxicological analysis of detoxified ZEA

The concluding study was commenced to know the toxicological safety of irradiation mediated detoxified ZEA, and it was assessed in RAW 264.7 cells by determining the cell viability, intracellular ROS molecules, MMP, nuclear damage, and caspase-3 activity.

The cell viability was assessed by two methods, i.e., MTT and live/dead dual staining assays. MTT assay is one of the widely used cell viability techniques in *in-vitro* studies and, which assess the cell viability based on metabolic activity of NAD(P)H-dependent oxidoreductase enzymes of cell (Fotakis and Timbrell, 2006; Venkataramana et al., 2014). The other cell viability technique, live/dead cell assay is dual staining technique comprising of calcein AM and ethidium homodimer-1 dyes. The calcein AM enter through the cell membrane and gets converted into fluorescent calcein by ubiquitous intracellular esterases of live cells and emits green fluorescence in live cells at an excitation and emission of 495 and 515 nm, respectively. Whereas, ethidium homodimer-1 enters through the damaged membrane of dead cells and strongly binds to nuclear material and produces red fluorescence in dead cells at an excitation of 495 nm and emission of 635 nm. The ethidium homodimer-1 is not a membrane permeable and excluded by membrane intact of live cells (Haugland et al., 1994; Kalagatur et al., 2017). In the present study, toxic effect of non-detoxified and irradiation mediation detoxified ZEA on cell viability was determined with respect to control. The MTT and live/dead cell assays concluded that cell viability was significantly (*p* < 0.05) decreased on treatment of non-detoxified ZEA (3 μg) related to control. While, cell viability was significantly (*p* < 0.05) high in cells treated with 5 and 10 kGy irradiation mediated detoxified ZEA (3 μg) related to non-detoxified ZEA (3 μg). In MTT assay, 14.10 ± 1.69, 64.05 ± 4.21, and 86.22 ± 2.73% of viable cells were observed in non-detoxified ZEA (3 μg), 5 kGy irradiation mediated detoxified ZEA (3 μg), and 10 kGy irradiation mediated detoxified ZEA (3 μg), respectively (Table 3). These results were well-supported by live/dead dual staining assay. The images of control cells, cells treated with non-detoxified ZEA (3 μg), and cells treated with 5 and 10 kGy irradiation mediated detoxified ZEA (3 μg) are shown in Supplementary Figure 4. The number of green fluorescent cells (live cells) in cells treated with non-detoxified ZEA (3 μg) was significantly less (*p* < 0.05) compared to control, and it was noticed as 11.29 ± 1.08% (Table 3). Whereas, 65.29 ± 3.37 and 89.67 ± 3.51% of live cells were observed in cells treated with irradiation mediated detoxified ZEA (3 μg) of 5 and 10 kGy, respectively (Table 3). A hundred percentage of live cells was not determined in irradiation mediated detoxified ZEA with respect to control, and this may be due to presence minute amount of non-detoxified ZEA. The percentage of ZEA reduction in 3 μg of ZEA was 41.89 ± 0.69%−47.02 ± 0.92% and 71.05 ± 0.81% at 5 and 10 kGy of irradiation, respectively and complete reduction of ZEA was not observed (Table 1). Therefore, 100% of live cells were not observed in cells treated with 5 and 10 kGy irradiation mediated detoxified ZEA. The study concluded that irradiation mediated detoxified ZEA was less toxic and safe compared to non-detoxified ZEA.

**Table 3:** Assessment of *in-vitro* toxicity of irradiation mediated detoxified ZEA and non-detoxified ZEA in RAW 264.7 cells for 12 h.

**Group**	**Test sample**	**Cell viability (%)**		**ROS (%)**	**MMP (%)**	**Caspase-3 (%)**
		**MTT**	**Live/dead**		
A	Control cells in 100 μL of DMEM devoid of FBS	100^a^	100^a^	100^a^	100^a^	100^a^
B	Cells treated with non-detoxified ZEA (3 μg) in 100 μL of DMEM devoid of FBS	14.10 ± 1.69^b^	11.29 ± 1.08^b^	321.6 ± 6.97^b^	22.82 ± 2.98^b^	226.4 ± 14.17^b^
C	Cells treated with detoxified ZEA (3 μg) of 5 kGy irradiated in 100 μL of DMEM devoid of FBS	64.05 ± 4.21^c^	65.29 ± 3.37^c^	173.9 ± 8.43^c^	60.47 ± 3.70^c^	162.1 ± 5.65^c^
D	Cells treated with detoxified ZEA (3 μg) of 10 kGy irradiated in 100 μL of DMEM devoid of FBS	86.22 ± 2.73^d^	89.67 ± 3.51^d^	128.7 ± 5.17^d^	81.90 ± 3.31^d^	114.2 ± 6.78^d^

*The statistical analysis was executed by Tukey's multiple comparison test and the columns with different alphabetic letters were statistically significant (p < 0.05) in respective study*.

Previous reports of Venkataramana et al. (2014), Kalagatur et al. (2017), Muthulakshmi et al. (2018), and Zheng et al. (2018) have revealed that ZEA induces the cell death through oxidative stress by generation of intracellular ROS molecules. The effect non-detoxified and irradiation mediated detoxified ZEA on generation of ROS molecules was determined by DCFH-DA staining. The DCFH-DA converts to non-fluorescent molecules through a deacetylation process over action of cellular esterases. Furthermore, oxidize to fluorescent 2′,7′-dichlorofluorescein molecules by intracellular ROS. The intensity of fluorescence is directly proportional to amount of ROS generated. In the present study, fluorescent images of control cells, cells treated with non-detoxified ZEA (3 μg), and cells treated with 5 and 10 kGy irradiation mediated detoxified ZEA (3 μg) are shown in Supplementary Figure 5. The fluorescence intensity and percentage of intracellular ROS molecules was high in cells treated with non-detoxified ZEA (3 μg) compared to control and observed as 321.6 ± 6.97% (*p* < 0.05). Another hand, cells treated with detoxified ZEA (3 μg) of 5 and 10 kGy irradiated have produced 173.9 ± 8.43% and 128.7 ± 5.17% of intracellular ROS molecules, respectively (*p* < 0.05) and the perceived fluorescence intensity was less compared to non-detoxified ZEA (3 μg) (Supplementary Figure 5 and Table 3). The small amount of intracellular ROS molecules was detected in cells treated with irradiation mediated detoxified ZEA due to presence of a smaller amount of non-detoxified ZEA. The results concluded that irradiation mediated detoxified ZEA has less capability to produce intracellular ROS molecules compared to non-detoxified ZEA and therefore, irradiation mediated detoxified ZEA was much safer compared to non-detoxified ZEA.

Also, earlier reports of Zhu et al. (2012), Venkataramana et al. (2014), and Kalagatur et al. (2017) have demonstrated that ZEA cause toxicity in cells by depletion of MMP levels. The effect of non-detoxified ZEA and irradiation mediated detoxified ZEA on MMP level was determined by rhodamine 123. The rhodamine 123 is a cell permeable dye and produce fluorescence by an appropriated act of metabolically active mitochondria at an excitation and emission of 511 and 534 nm, respectively and, which is used to consider as an indicator for MMP. The depletion in MMP could halt ATP synthesis and trigger death by an apoptosis process (Hussain et al., 2005). In the present study, MMP levels in cells were depleted on exposure of non-detoxified ZEA (3 μg) compared to control and noticed as 22.82 ± 2.98%. Remarkably, MMP levels were significantly high in cells treated with irradiation mediated detoxified ZEA compared to non-detoxified ZEA, and it was determined as 60.47 ± 3.70% and 81.90 ± 3.31% in cells treated with 5 and 10 kGy irradiation mediated detoxified ZEA (3 μg), respectively (Table 3). Correspondingly, fluorescent images of MMP analysis are shown in Supplementary Figure 6. A low fluorescence intensity was noticed in cells treated with non-detoxified ZEA (3 μg) due to depletion of MMP levels compared to control cells. Moreover, high intensity of fluorescence was perceived in cells treated with 5 and 10 kGy irradiation mediated detoxified ZEA (3 μg) related to non-detoxified ZEA (3 μg).

Many *in-vitro* studies have demonstrated that ZEA induces the cell death through an apoptosis process by introducing nuclear damage and elevating the activity of caspase-3 (Zhu et al., 2012; Venkataramana et al., 2014; Wang et al., 2014; Tatay et al., 2016; Kalagatur et al., 2017; Zheng et al., 2018). The DAPI staining is relied upon the principle that intact DNA holds a well-organized association with protein matrix of nucleus and appears round and intact in a center of the cell. While, cells on exposure to toxic substances produce fragmented and disrupted nuclear material as a result intact DNA assembly with protein matrix at a center of cell tends to lose and could be noticed by bright fluorescent intensity and leakage of nuclear material from cell, which is a hallmark of apoptosis (Venkataramana et al., 2014). In the present study, DAPI fluorescent images of control cells, cells treated with non-detoxified ZEA (3 μg), and cells treated with 5 and 10 kGy irradiation mediated detoxified ZEA (3 μg) are shown in Supplementary Figure 7. The nuclear damage, i.e., bright fluorescent and leaky nuclei were noticed in cells treated with non-detoxified ZEA (3 μ) g), and the nuclear damage were much less perceived in cells treated with 5 and 10 kGy irradiation mediated detoxified ZEA (3 μg). To conclude, effect of non-detoxified ZEA and irradiation mediated detoxified ZEA on apoptosis was assessed by measuring caspase-3 activity. The caspase-3 is a member of caspase family and its successive activation of caspases plays a vital role in the accomplishment of cellular apoptosis (Chen et al., 1998; Porter and Jänicke, 1999). In the present study, the caspase-3 activity was high in non-detoxified ZEA (3 μg) compared to control (100%) and noticed as 226.4 ± 14.17% (*p* < 0.05). Whereas, cells exposed with detoxified ZEA (3 μg) of 5 and 10 kGy irradiated have exhibited 162.1 ± 5.65 and 114.2 ± 6.78% of caspase-3 activity, respectively (Table 3). The study showed that caspase-3 activity was less elevated in irradiation mediated detoxified ZEA compared to non-detoxified ZEA. The slight activity of caspase-3 was determined in irradiation mediated detoxified ZEA due to the presence of smaller amounts of non-detoxified ZEA. The results were in accordance with the analysis of cell viability, intracellular ROS molecules, MMP, and nuclear damage. The outcome from the study clearly evidenced that detoxification of ZEA using irradiation produce non-toxic by-products, and this is the first report. However, further studies should be carried out on identification and purification of radiolytic products of ZEA to propose the detailed toxic feature of detoxified ZEA.

### Quality assessment of fruit juices treated with irradiation

Effect of different irradiation doses on quality of fruit juice was assessed by considering various parameters, i.e., sensory (appearance, aroma, consistency, and taste), pH, acidity, total soluble solids, total phenolic and flavonoid content, and total antioxidant activity (Tables 4–6).

**Table 4:** Quality assessment of orange fruit juice treated with different doses of irradiation.

**Quality parameter**	**Irradiation dose**				
	**0 kGy (Control)**	**2.5 kGy**	**5 kGy**	**7.5 kGy**	**10 kGy**
1.Sensory attributes				
A. Appearance^©^	7.75 ± 0.16	7.65 ± 0.32^#^	7.34 ± 0.28^#^	6.97 ± 0.14^#^	6.50 ± 0.21^*^
B. Aroma^©^	8.31 ± 0.23	8.23 ± 0.37^#^	7.84 ± 0.31^#^	7.15 ± 0.12^#^	6.20 ± 0.24^*^
C. Consistency^©^	7.59 ± 0.18	7.55 ± 0.15^#^	7.52 ± 0.26^#^	7.48 ± 0.11^#^	7.14 ± 0.19^#^
D. Taste^©^	8.18 ± 0.41	7.84 ± 0.24^#^	7.51 ± 0.33^#^	6.73 ± 0.16^#^	6.07 ± 0.30^*^
E. Overall acceptability^$^	8.08 ± 0.27	7.8 ± 0.19^#^	7.51 ± 0.22^#^	6.94 ± 0.38^#^	6.57 ± 0.21^*^
2. Total soluble solids (°Brix)	12.50 ± 0.79	12.4 ± 0.84^#^	12.6 ± 0.69^#^	12.5 ± 0.31^#^	12.6 ± 0.55^#^
3. Acidity (% citric acid)	0.62 ± 0.04	0.62 ± 0.06^#^	0.63 ± 0.02^#^	0.65 ± 0.06^#^	0.68 ± 0.04^#^
4. pH	3.78 ± 0.14	3.78 ± 0.27^#^	3.77 ± 0.26^#^	3.75 ± 0.11^#^	3.14 ± 0.14^#^
5. Total phenolic content (mg GAE/mL)	0.89 ± 0.07	0.83 ± 0.07^#^	0.76 ± 0.05^#^	0.71 ± 0.04^#^	0.67 ± 0.05^#^
6. Total flavonoid content (mg CE/mL)	0.51 ± 0.02	0.49 ± 0.03^#^	0.44 ± 0.04^#^	0.43 ± 0.07^#^	0.32 ± 0.01^*^
7. Total antioxidant activity (% inhibition of DPPH radical)	31.65 ± 1.18	30.44 ± 0.94^#^	28.91 ± 0.77^#^	27.1 ± 0.59^#^	26.05 ± 1.01^*^

Quality assessment of irradiated fruit juices was compared with control by Dunnett's test applying software GraphPad Prism trial version 7. The p < 0.05 was considered as statistically significant and represented as ^*^. Whereas, p > 0.05 was considered as statistically not significant and represented as #.

© *1: Extremely poor. 2: Very poor. 3: Poor. 4: Fair above poor. 5: Fair. 6: Good above fair. 7: Good. 8: Very good. 9: Excellent*.

$ *1: Dislike extremely. 2: Dislike very much. 3: Dislike moderately. 4: Dislike slightly. 5: Neither like nor dislike. 6: Like slightly. 7: Like moderately. 8: Like very much. 9: Like extremely*.

**Table 5:** Quality assessment of pineapple fruit juice treated with different doses of irradiation.

**Quality parameter**	**Irradiation dose**				
	**0 kGy (Control)**	**2.5 kGy**	**5 kGy**	**7.5 kGy**	**10 kGy**
1.Sensory attributes				
A. Appearance^©^	8.11 ± 0.49	8.05 ± 0.26^#^	7.81 ± 0.33^#^	7.59 ± 0.25^#^	7.12 ± 0.27^*^
B. Aroma^©^	8.56 ± 0.27	8.25 ± 0.18^#^	7.9 ± 0.27^#^	7.32 ± 0.31^#^	6.91 ± 0.22^*^
C. Consistency^©^	7.60 ± 0.14	7.5 ± 0.29^#^	7.35 ± 0.12^#^	7.22 ± 0.16^#^	7.00 ± 0.24^#^
D. Taste^©^	8.60 ± 0.38	8.25 ± 0.20^#^	8.07 ± 0.27^#^	7.82 ± 0.31^#^	7.24 ± 0.23^*^
E. Overall acceptability^$^	8.71 ± 0.46	8.33 ± 0.41^#^	8.01 ± 0.29^#^	7.44 ± 0.37^#^	6.82 ± 0.22^*^
2. Total soluble solids (°Brix)	14.8 ± 0.39	14.8 ± 0.64^#^	14.9 ± 0.41^#^	14.8 ± 0.27^#^	15.1 ± 0.83^#^
3. Acidity (% citric acid)	0.51 ± 0.02	0.52 ± 0.05^#^	0.54 ± 0.04^#^	0.55 ± 0.02^#^	0.57 ± 0.04^#^
4. pH	3.95 ± 0.17	3.93 ± 0.18^#^	3.90 ± 0.22^#^	3.90 ± 0.16^#^	3.88 ± 0.18^#^
5. Total phenolic content (mg GAE/mL)	0.76 ± 0.07	0.72 ± 0.06^#^	0.62 ± 0.04^#^	0.59 ± 0.03^#^	0.55 ± 0.06^*^
6. Total flavonoid content (mg CE/mL)	0.37 ± 0.04	0.36 ± 0.08^#^	0.34 ± 0.04^#^	0.31 ± 0.07^#^	0.18 ± 0.00^*^
7. Total antioxidant activity (% inhibition of DPPH radical)	22.59 ± 0.84	22.1 ± 1.09^#^	20.15 ± 0.69^#^	19.48 ± 0.91^#^	18.22 ± 0.58^*^

Quality assessment of irradiated fruit juices was compared with control by Dunnett's test applying software GraphPad Prism trial version 7. The p < 0.05 was considered as statistically significant and represented as ^*^. Whereas, p > 0.05 was considered as statistically not significant and represented as #.

© *1: Extremely poor. 2: Very poor. 3: Poor. 4: Fair above poor. 5: Fair. 6: Good above fair. 7: Good. 8: Very good. 9: Excellent*.

$ *1: Dislike extremely. 2: Dislike very much. 3: Dislike moderately. 4: Dislike slightly. 5: Neither like nor dislike. 6: Like slightly. 7: Like moderately. 8: Like very much. 9: Like extremely*.

**Table 6:** Quality assessment of tomato juice treated with different doses of irradiation.

**Quality parameter**	**Irradiation dose**				
	**0 kGy (Control)**	**2.5 kGy**	**5 kGy**	**7.5 kGy**	**10 kGy**
1.Sensory attributes				
A. Appearance^©^	7.21 ± 0.29	7.02 ± 0.14^#^	6.87 ± 0.35^#^	6.51 ± 0.41^#^	6.31 ± 0.22^*^
B. Aroma^©^	7.82 ± 0.34	7.60 ± 0.16^#^	7.25 ± 0.11^#^	6.88 ± 0.26^#^	6.52 ± 0.19^*^
C. Consistency^©^	7.50 ± 0.29	7.45 ± 0.11^#^	7.38 ± 0.19^#^	7.24 ± 0.19^#^	7.20 ± 0.24^#^
D. Taste^©^	7.11 ± 0.46	7.00 ± 0.33^#^	6.80 ± 0.26^#^	6.42 ± 0.24^#^	6.00 ± 0.37^*^
E. Overall acceptability^$^	7.64 ± 0.37	7.52 ± 0.33^#^	7.01 ± 0.19^#^	6.72 ± 0.28^#^	6.31 ± 0.31^*^
2. Total soluble solids (°Brix)	5.20 ± 0.22	5.16 ± 0.27^#^	5.21 ± 0.06^#^	5.32 ± 0.09^#^	5.30 ± 0.14^#^
3. Acidity (% citric acid)	0.65 ± 0.03^#^	0.66 ± 0.05^#^	0.69 ± 0.03^#^	0.70 ± 0.02^#^	0.72 ± 0.04^#^
4. pH	3.72 ± 0.21^#^	3.72 ± 0.37^#^	3.70 ± 0.24^#^	3.69 ± 0.19^#^	3.65 ± 0.27^#^
5. Total phenolic content (mg GAE/mL)	0.81 ± 0.05	0.78 ± 0.02^#^	0.76 ± 0.03^#^	0.72 ± 0.04^#^	0.7 ± 0.02^*^
6. Total flavonoid content (mg CE/mL)	0.39 ± 0.01	0.38 ± 0.04^#^	0.36 ± 0.07^#^	0.34 ± 0.02^#^	0.18 ± 0.01^*^
7. Total antioxidant activity (% inhibition of DPPH radical)	29.83 ± 0.89	28.15 ± 0.73^#^	26.72 ± 1.10^#^	25.39 ±.81^#^	24.15 ± 0.94^*^

Quality assessment of irradiated fruit juices was compared with control by Dunnett's test applying software GraphPad Prism trial version 7. The p < 0.05 was considered as statistically significant and represented as ^*^. Whereas, p > 0.05 was considered as statistically not significant and represented as #.

© *1: Extremely poor. 2: Very poor. 3: Poor. 4: Fair above poor. 5: Fair. 6: Good above fair. 7: Good. 8: Very good. 9: Excellent*.

$ *1: Dislike extremely. 2: Dislike very much. 3: Dislike moderately. 4: Dislike slightly. 5: Neither like nor dislike. 6: Like slightly. 7: Like moderately. 8: Like very much. 9: Like extremely*.

The sensory evaluation showed that irradiation doses of 2.5, 5, and 7.5 kGy have no significant effect on quality of fruit juices compared to control. While, 10 kGy of irradiation has produced significant changes in sensory attributes except consistency of fruit juices compared to control. Subsequently, overall acceptability of fruit juices has significant affected at high dose of 10 kGy compared to control. The observed sensory results could be due to production of off-flavor and off-color in the fruit juices during irradiation processing (Yun et al., 2010).

Further, control and irradiation treated fruit juices were analyzed for total soluble solids and results revealed that irrespective of radiation doses, total soluble solids have shown no significant difference related to control. In support of our results, earlier reports of Arjeh et al. (2015) and Naresh et al. (2015) have reported that irradiation dose of 6 and 3 kGy not produced significant changes in total soluble solids of cherry and mango juices, respectively. On the other hand, acidity and pH were correspondingly increased and decreased in fruit juices upon irradiation and insignificant changes were noticed in fruit juices at all irradiation doses related to control. In support of these results, Youssef et al. (2002) and Harder et al. (2009) have reported a slight rise in acidity and reduction in pH of mango pulp and nectar of kiwi fruits, respectively and concluded that may be due to inactivation of citric acid cleaving enzyme.

Also, total phenolic and flavonoid contents of fruit juices were decreased upon irradiation and significant changes were observed at 10 kGy compared to control. The total phenolic and flavonoid contents were decreased in irradiated fruit juices and it could be due to degradative action of irradiation on phenolic and flavonoid contents (Najafabadi et al., 2017). Likewise, total antioxidant activity of fruit juices was decreased upon irradiation and significant changes were noticed in 10 kGy compared to control. The antioxidant potential of plant derived products mainly depend on its phenolic and flavonoid contents (George et al., 2016; Muniyandi et al., 2017). In this study, total phenolic and flavonoid contents were decreased in irradiated fruit juices compared to control. Therefore, might be antioxidant activity of fruit juices was decreased upon irradiation compared to control.

Overall, study determined that irradiation of fruit juices with high doses minimally alters the quality of fruit juices. However, irradiation enhances the microbiological safety and prolongs the shelf life of food products. Thus, WHO and FAO has specified that irradiation of food products up to 25 kGy are safe and recognized as suitable decontamination technique in agriculture and food industry (FAO/IAEA/WHO, 1999).

## Conclusion

In the present study, detoxification efficacy of irradiation on ZEA in water and fruit juice was assessed by CCD of RSM statistical program. The independent factors (dose of irradiation and concentration of zearalenone) had a positive significance on the response factor (percentage of ZEA reduction). The RSM study concluded that dose of irradiation and concentration of zearalenone were the determinant factors for detoxification of ZEA. The toxic effects of detoxified ZEA were studied under *in-vitro* conditions. The irradiation mediated detoxified ZEA has exhibited less toxicity compared to non-detoxified ZEA. The results confirmed that the toxicity of ZEA was decreased with irradiation treatment. To reveal the precise process of ZEA detoxification, further research is needed on extraction, purification, and structural elucidation of radiolytic products of ZEA. In conclusion, due to its rapidity and effectiveness on detoxification of ZEA, irradiation could be a potential food processing technique in the agriculture and food industry. However, irradiation of fruit juices with high dose of 10 kGy has minimally altered the quality of fruit juices. Nevertheless, irradiation process should carry out with well-directed standard operating procedures (SOPs) in approved laboratories as per FAO/IAEA/WHO.

## Author contributions

NK and VM designed the work. NK, JK, and VM executed the work, analyzed data, and drafted the results. All authors have approved the final version of the manuscript.

### Conflict of interest statement

The authors declare that the research was conducted in the absence of any commercial or financial relationships that could be construed as a potential conflict of interest.

## Supplementary material

Supplementary Table S1 Experimental range, levels, mean, and standard deviation of independent variables, i.e. zearalenone (ZEA) and irradiation.

Supplementary Table S2 HPLC conditions for determination of zearalenone (ZEA).

Supplementary Table S3 ANOVA for percentage of zearalenone (ZEA) reduction in distilled water.

Supplementary Table S4 ANOVA for percentage of zearalenone (ZEA) reduction in orange juice.

Supplementary Table S5 ANOVA for percentage of zearalenone (ZEA) reduction in pineapple juice.

Supplementary Table S6 ANOVA for percentage of zearalenone (ZEA) reduction in tomato juice.

Supplementary Table S7 Sequential model and regression coefficients of optimized designs.

Supplementary Figure S1 Normal plot of residuals for detoxification effect of irradiation on zearalenone (ZEA) in **(A)** distilled water, **(B)** orange juice, **(C)** pineapple juice, and **(D)** tomato juice.

Supplementary Figure S2 Box-cox plots for detoxification effect of irradiation on zearalenone (ZEA) in **(A)** distilled water, **(B)** orange juice, **(C)** pineapple juice, and **(D)** tomato juice.

Supplementary Figure S3 Actual versus predicted plots for detoxification effect of irradiation on zearalenone (ZEA) in **(A)** distilled water, **(B)** orange juice, **(C)** pineapple juice, and **(D)** tomato juice.

Supplementary Figure S4 Assessment of toxic effect of non-detoxified and irradiation mediated detoxified zearalenone (ZEA) on cell viability in RAW 264.7 cells for 12 h by live/dead dual staining technique.

Supplementary Figure S5 Assessment of effect of non-detoxified and irradiation mediated detoxified zearalenone (ZEA) on generation of intracellular reactive oxygen species (ROS) in RAW 264.7 cells for 12 h by DCFH-DA staining.

Supplementary Figure S6 Assessment of effect of non-detoxified and irradiation mediated detoxified zearalenone (ZEA) on mitochondrial membrane potential (MMP) in RAW 264.7 cells for 12 h by rhodamine 123 staining.

Supplementary Figure S7 Assessment of effect of non-detoxified and irradiation mediated detoxified zearalenone (ZEA) on nuclear damage in RAW 264.7 cells for 12 h by DAPI staining.
