# Supplementary material for: Assessment of Detoxification Efficacy of Irradiation on Zearalenone Mycotoxin in Various Fruit Juices by Response Surface Methodology and Elucidation of Its in-vitro Toxicity
Source: Front Microbiol. 2018 Nov 30;9:2937. doi: 10.3389/fmicb.2018.02937 (PMC6284055; doi:10.3389/fmicb.2018.02937)
Supplement: Supplementary Table S1 — Experimental range, levels, mean, and standard deviation of independent variables, i.e. zearalenone (ZEA) and irradiation. [file Table_1.DOCX]

**Supplementary Table 1:** Experimental range, levels, mean, and standard deviation of independent variables, i.e. zearalenone (ZEA) and irradiation.

| Factor | Name | Units | Type | Range | |  | Levels | | | | |  | Mean | Standard deviation |
| --- | --- | --- | --- | --- | --- | --- | --- | --- | --- | --- | --- | --- | --- | --- |
|  |  |  |  | Minimum | Maximum |  | -α | -1 | 0 | 1 | α |  |  |  |
| A | ZEA | µg | numeric | 1 | 5 |  | 1 | 1.58 | 3.00 | 4.41 | 5 |  | 3.00 | 1.10 |
| B | Irradiation | kGy | numeric | 0 | 10 |  | 0 | 1.46 | 5.00 | 8.53 | 10 |  | 5.00 | 2.77 |
