# Supplementary material for: Assessment of Detoxification Efficacy of Irradiation on Zearalenone Mycotoxin in Various Fruit Juices by Response Surface Methodology and Elucidation of Its in-vitro Toxicity
Source: Front Microbiol. 2018 Nov 30;9:2937. doi: 10.3389/fmicb.2018.02937 (PMC6284055; doi:10.3389/fmicb.2018.02937)
Supplement: Supplementary Table S2 — HPLC conditions for determination of zearalenone (ZEA). [file Table_2.DOCX]

**Supplementary Table 2:** HPLC conditions for determination of zearalenone (ZEA).

| Column | Phenomenex C18, 5 μm thickness, 250 mm × ID of 4.6 mm length, and pore size of 100Å |
| --- | --- |
| Column temperature | Room temperature |
| Sample injection volume | 25 µL |
| Mobile phase | Acetonitrile and water (1:1, v/v) |
| Flow rate of mobile phase | 1 mL/min |
| Runtime | 15 min |
| Detector | Fluorescence |
| Analysis phase | Reverse phase |
| Excitation wavelength | 334 nm |
| Emission wavelength | 450 nm |
