# Supplementary material for: Assessment of Detoxification Efficacy of Irradiation on Zearalenone Mycotoxin in Various Fruit Juices by Response Surface Methodology and Elucidation of Its in-vitro Toxicity
Source: Front Microbiol. 2018 Nov 30;9:2937. doi: 10.3389/fmicb.2018.02937 (PMC6284055; doi:10.3389/fmicb.2018.02937)
Supplement: Supplementary Table S3 — ANOVA for percentage of zearalenone (ZEA) reduction in distilled water. [file Table_3.DOCX]

**Supplementary Table 3:** ANOVA for percentage of zearalenone (ZEA) reduction in distilled water.

| Source | Sum of squares | Degree of freedom (df) | Mean square | F value | *p*-value Prob > F |
| --- | --- | --- | --- | --- | --- |
| Model | 6527.99 | 5 | 1305.60 | 297.27 | < 0.0001 significant |
| A-Zearalenone | 1293.18 | 1 | 129.18 | 294.45 | < 0.0001 |
| B-Gamma radiation | 4855.55 | 1 | 4855.55 | 1105.57 | < 0.0001 |
| AB | 63.12 | 1 | 63.12 | 14.37 | < 0.0068 |
| A^2^ | 124.53 | 1 | 124.53 | 28.35 | 0.0011 |
| B^2^ | 150.51 | 1 | 150.51 | 34.27 | 0.0006 |
| Residual | 30.74 | 7 | 4.39 |  |  |
| Lack of Fit | 8.89 | 3 | 2.96 | 0.54 | 0.6787 not significant |
| Pure Error | 21.85 | 4 | 5.46 |  |  |
| Cor Total | 6558.73 | 12 |  |  |  |
