# Supplementary material for: Assessment of Detoxification Efficacy of Irradiation on Zearalenone Mycotoxin in Various Fruit Juices by Response Surface Methodology and Elucidation of Its in-vitro Toxicity
Source: Front Microbiol. 2018 Nov 30;9:2937. doi: 10.3389/fmicb.2018.02937 (PMC6284055; doi:10.3389/fmicb.2018.02937)
Supplement: Supplementary Table S4 — ANOVA for percentage of zearalenone (ZEA) reduction in orange juice. [file Table_4.DOCX]

**Supplementary Table 4:** ANOVA for percentage of zearalenone (ZEA) reduction in orange juice.

| Source | Sum of squares | Degree of freedom (df) | Mean square | F value | *p*-value Prob > F |
| --- | --- | --- | --- | --- | --- |
| Model | 6281.096 | 5 | 1256.21 | 455.44 | < 0.0001 significant |
| A-Zearalenone | 1251.102 | 1 | 1251.10 | 453.59 | < 0.0001 |
| B-Gamma radiation | 4676.674 | 1 | 4676.67 | 1695.54 | < 0.0001 |
| AB | 66.5856 | 1 | 66.58 | 24.14 | 0.0017 |
| A^2^ | 113.4994 | 1 | 113.49 | 41.14 | 0.0004 |
| B^2^ | 135.9515 | 1 | 135.95 | 49.28 | 0.0002 |
| Residual | 19.30747 | 7 | 2.75 |  |  |
| Lack of Fit | 3.454151 | 3 | 1.15 | 0.29 | 0.8311 not significant |
| Pure Error | 15.85332 | 4 | 3.96 |  |  |
| Cor Total | 6300.403 | 12 |  |  |  |
