# Supplementary material for: Assessment of Detoxification Efficacy of Irradiation on Zearalenone Mycotoxin in Various Fruit Juices by Response Surface Methodology and Elucidation of Its in-vitro Toxicity
Source: Front Microbiol. 2018 Nov 30;9:2937. doi: 10.3389/fmicb.2018.02937 (PMC6284055; doi:10.3389/fmicb.2018.02937)
Supplement: Supplementary Table S5 — ANOVA for percentage of zearalenone (ZEA) reduction in pineapple juice. [file Table_5.DOCX]

**Supplementary Table 5:** ANOVA for percentage of zearalenone (ZEA) reduction in pineapple juice.

| Source | Sum of squares | Degree of freedom (df) | Mean square | F value | *p*-value Prob > F |
| --- | --- | --- | --- | --- | --- |
| Model | 6335.203 | 5 | 1267.04 | 444.39 | < 0.0001 significant |
| A-Zearalenone | 1184.352 | 1 | 1184.35 | 415.39 | < 0.0001 |
| B-Gamma radiation | 4874.232 | 1 | 4874.23 | 1709.56 | < 0.0001 |
| AB | 51.76803 | 1 | 51.76 | 18.15 | 0.0037 |
| A^2^ | 97.34407 | 1 | 97.34 | 34.14 | 0.0006 |
| B^2^ | 98.17858 | 1 | 98.17 | 34.43 | 0.0006 |
| Residual | 19.95809 | 7 | 2.85 |  |  |
| Lack of Fit | 4.000773 | 3 | 1.33 | 0.33 | 0.8026 not significant |
| Pure Error | 15.95732 | 4 | 3.98 |  |  |
| Cor Total | 6355.161 | 12 |  |  |  |
