# Supplementary material for: Assessment of Detoxification Efficacy of Irradiation on Zearalenone Mycotoxin in Various Fruit Juices by Response Surface Methodology and Elucidation of Its in-vitro Toxicity
Source: Front Microbiol. 2018 Nov 30;9:2937. doi: 10.3389/fmicb.2018.02937 (PMC6284055; doi:10.3389/fmicb.2018.02937)
Supplement: Supplementary Table S6 — ANOVA for percentage of zearalenone (ZEA) reduction in tomato juice. [file Table_6.DOCX]

**Supplementary Table 6:** ANOVA for percentage of zearalenone (ZEA) reduction in tomato juice.

| Source | Sum of squares | Degree of freedom (df) | Mean square | F value | *p*-value Prob > F |
| --- | --- | --- | --- | --- | --- |
| Model | 6230.236 | 5 | 1246.04 | 345.47 | < 0.0001 significant |
| A-Zearalenone | 1103.685 | 1 | 1103.68 | 306.00 | < 0.0001 |
| B-Gamma radiation | 4817.422 | 1 | 4817.42 | 1335.66 | < 0.0001 |
| AB | 48.09423 | 1 | 48.09 | 13.33 | 0.0082 |
| A^2^ | 120.901 | 1 | 120.90 | 33.52 | 0.0007 |
| B^2^ | 106.1413 | 1 | 106.14 | 29.42 | 0.0010 |
| Residual | 25.24736 | 7 | 3.60 |  |  |
| Lack of Fit | 6.840039 | 3 | 2.28 | 0.49 | 0.7048 not significant |
| Pure Error | 18.40732 | 4 | 4.60 |  |  |
| Cor Total | 6255.483 | 12 |  |  |  |
