# Supplementary material for: Assessment of Detoxification Efficacy of Irradiation on Zearalenone Mycotoxin in Various Fruit Juices by Response Surface Methodology and Elucidation of Its in-vitro Toxicity
Source: Front Microbiol. 2018 Nov 30;9:2937. doi: 10.3389/fmicb.2018.02937 (PMC6284055; doi:10.3389/fmicb.2018.02937)
Supplement: Supplementary Table S7 — Sequential model and regression coefficients of optimized designs. [file Table_7.DOCX]

**Supplementary Table 7:** Sequential model and regression coefficients of optimized designs.

| Term model | Responses (percentage of zearalenone reduction) | | | |
| --- | --- | --- | --- | --- |
|  | Distilled water | Orange juice | Pineapple juice | Tomato juice |
| F Value | 297.27 | 455.45 | 1267.04 | 1246.05 |
| *p* > F | < 0.0001 | < 0.0001 | < 0.0001 | < 0.0001 |
| Mean | 44.24 | 43.11 | 42.60 | 43.10 |
| Standard deviation | 2.10 | 1.66 | 1.69 | 1.90 |
| C V % | 4.74 | 3.85 | 3.96 | 4.41 |
| R squared | 0.9953 | 0.9969 | 0.9969 | 0.9960 |
| Adjusted R squared | 0.9920 | 0.9947 | 0.9946 | 0.9931 |
| Predicted R squared | 0.9852 | 0.9922 | 0.9916 | 0.9876 |
| Adequate precision | 59.734 | 73.933 | 72.227 | 63.967 |
| Model | Quadratic | Quadratic | Quadratic | Quadratic |
