# Supplementary figures and images for: Assessment of Detoxification Efficacy of Irradiation on Zearalenone Mycotoxin in Various Fruit Juices by Response Surface Methodology and Elucidation of Its in-vitro Toxicity
Source: Front Microbiol. 2018 Nov 30;9:2937. doi: 10.3389/fmicb.2018.02937 (PMC6284055; doi:10.3389/fmicb.2018.02937)

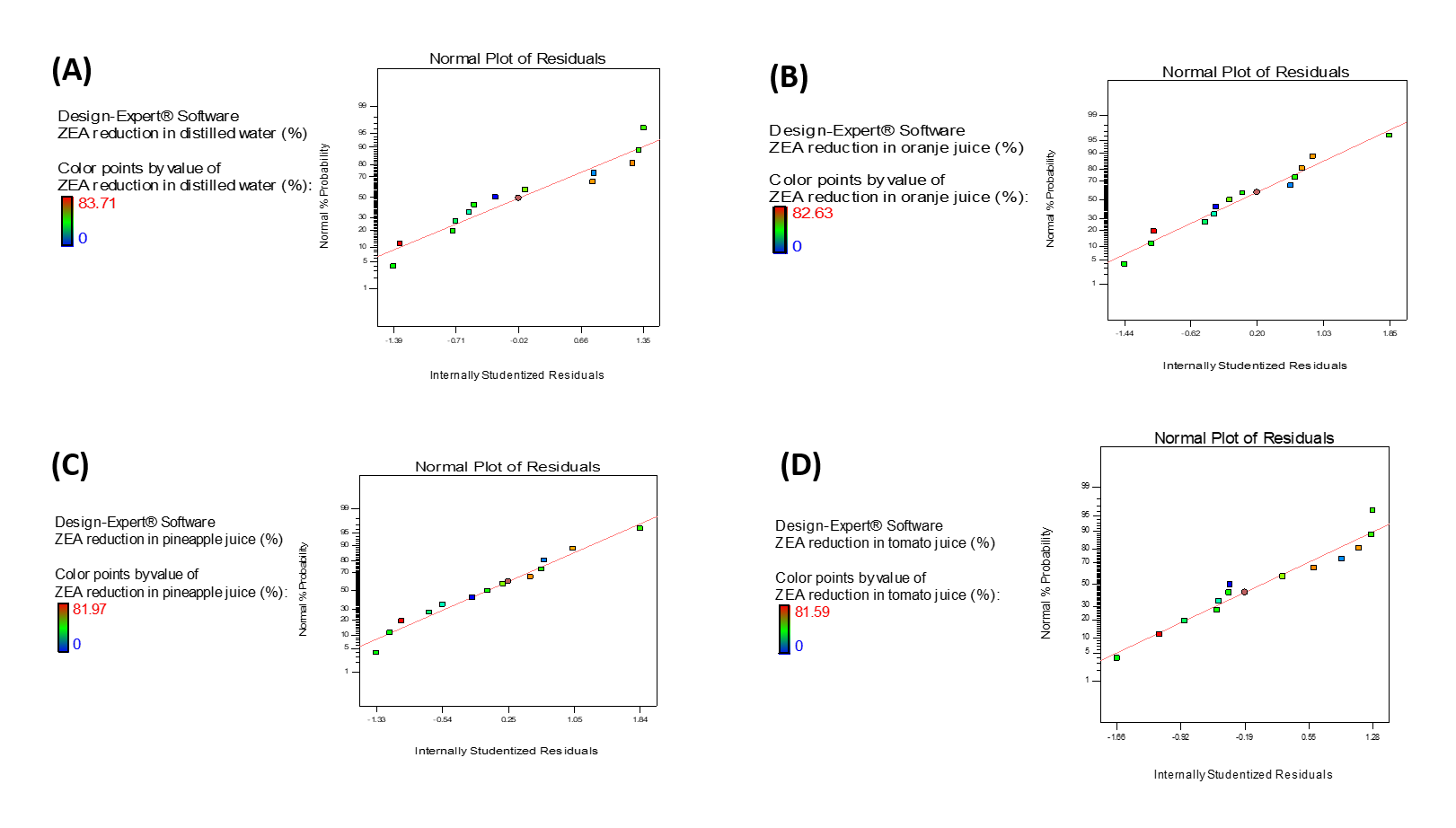

Supplement: Supplementary Figure S1 — Normal plot of residuals for detoxification effect of irradiation on zearalenone (ZEA) in (A) distilled water, (B) orange juice, (C) pineapple juice, and (D) tomato juice. [file Image_1.TIF]

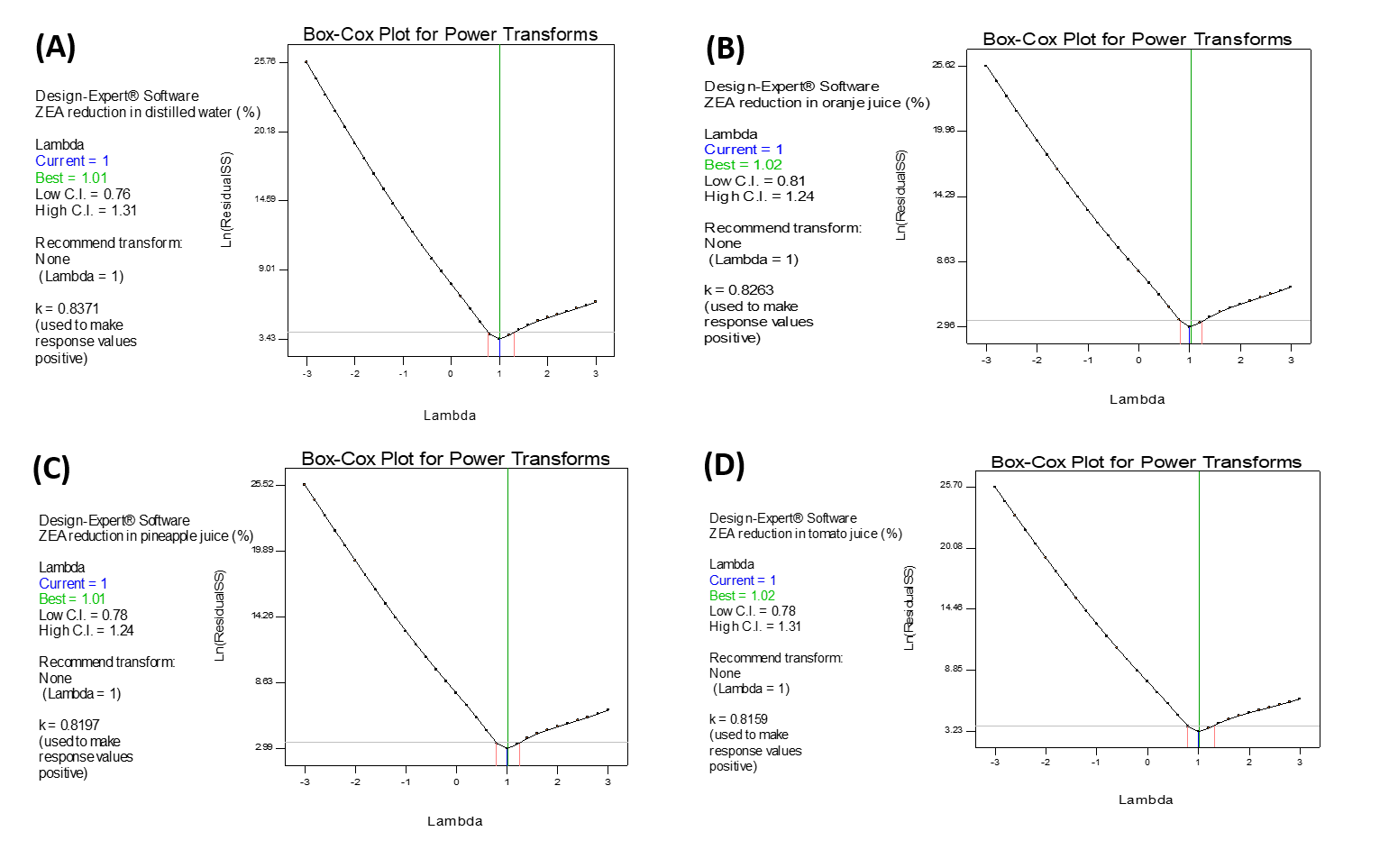

Supplement: Supplementary Figure S2 — Box-cox plots for detoxification effect of irradiation on zearalenone (ZEA) in (A) distilled water, (B) orange juice, (C) pineapple juice, and (D) tomato juice. [file Image_2.TIF]

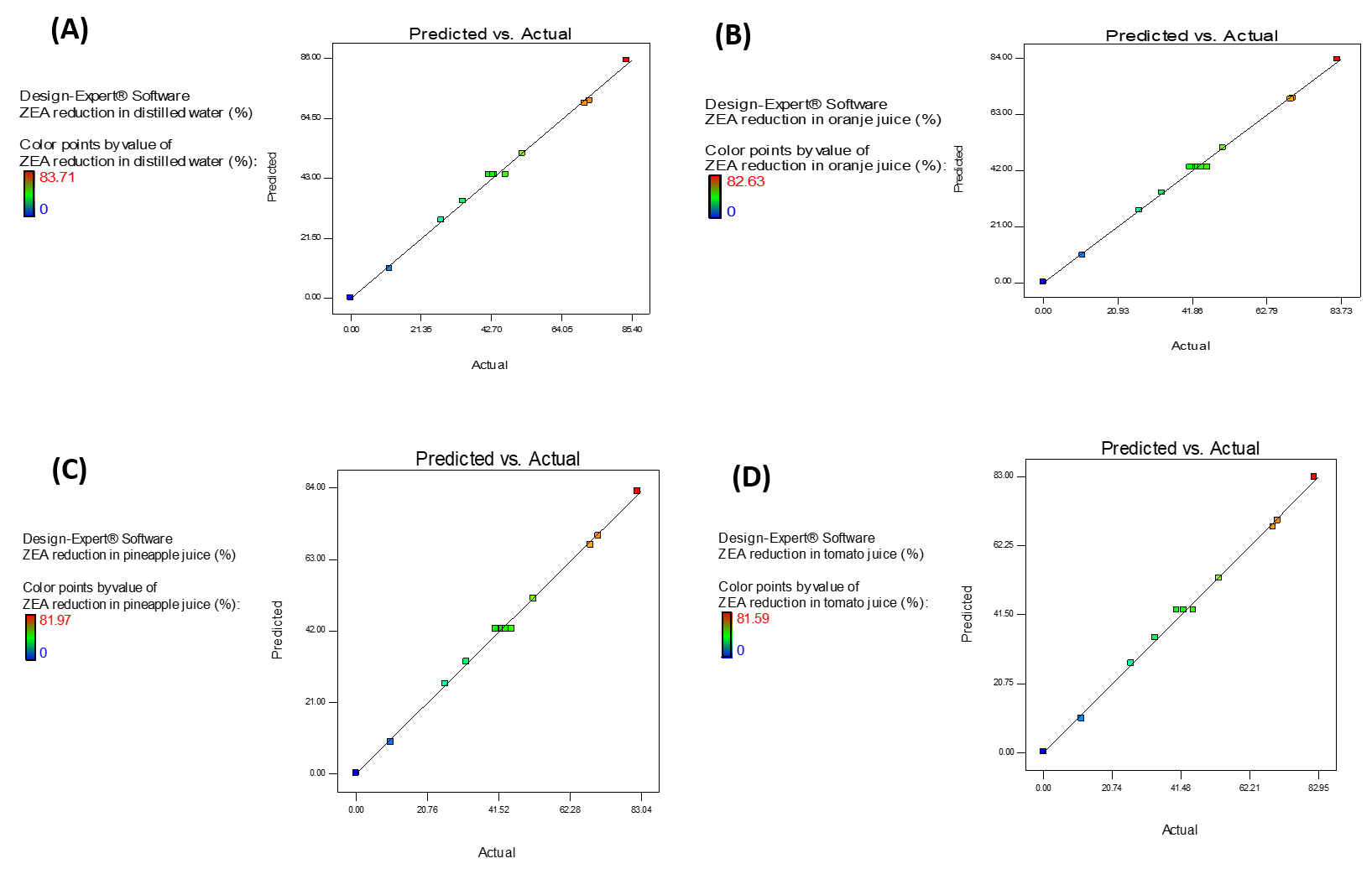

Supplement: Supplementary Figure S3 — Actual versus predicted plots for detoxification effect of irradiation on zearalenone (ZEA) in (A) distilled water, (B) orange juice, (C) pineapple juice, and (D) tomato juice. [file Image_3.TIF]

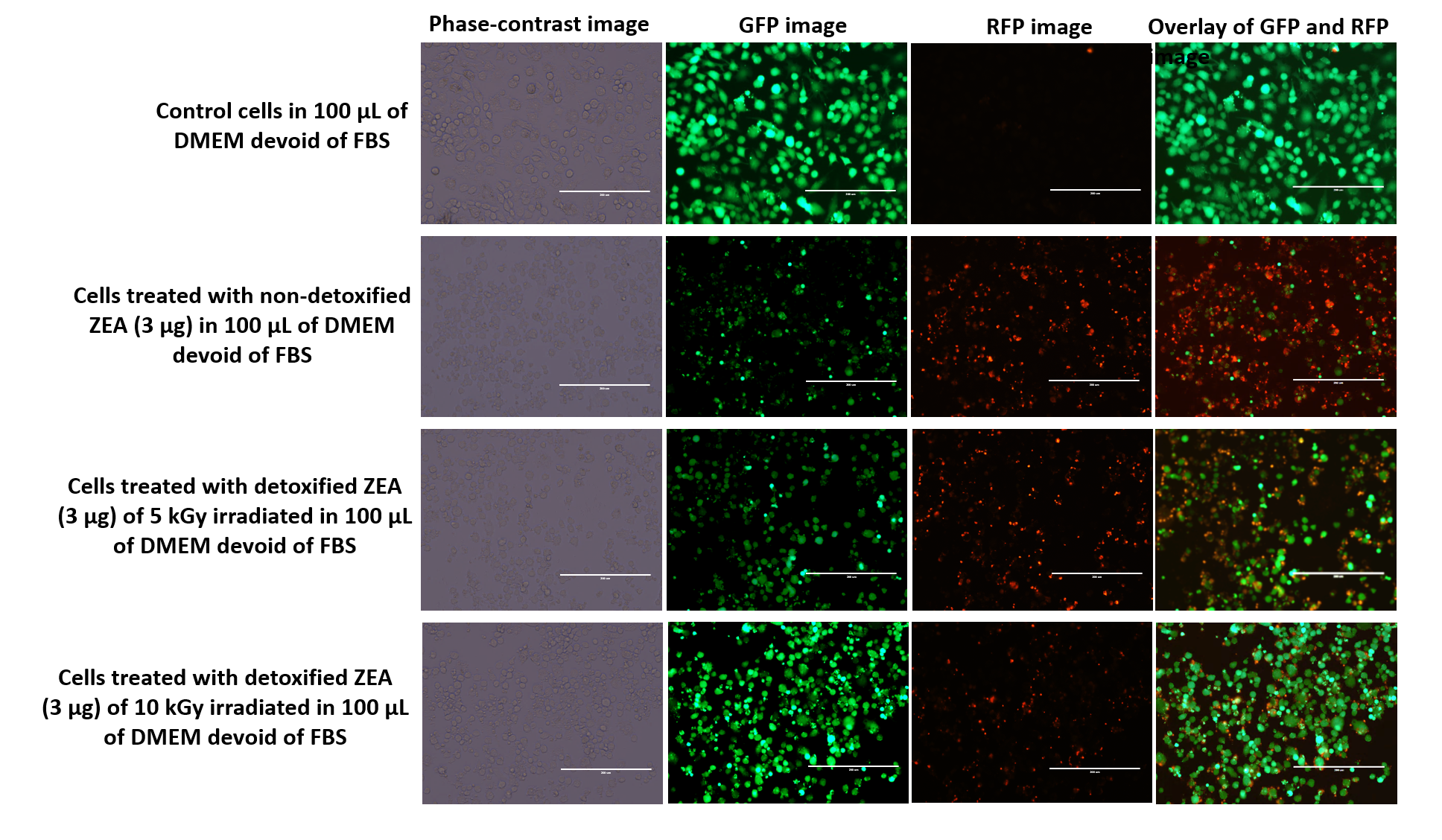

Supplement: Supplementary Figure S4 — Assessment of toxic effect of non-detoxified and irradiation mediated detoxified zearalenone (ZEA) on cell viability in RAW 264.7 cells for 12 h by live/dead dual staining technique. [file Image_4.TIF]

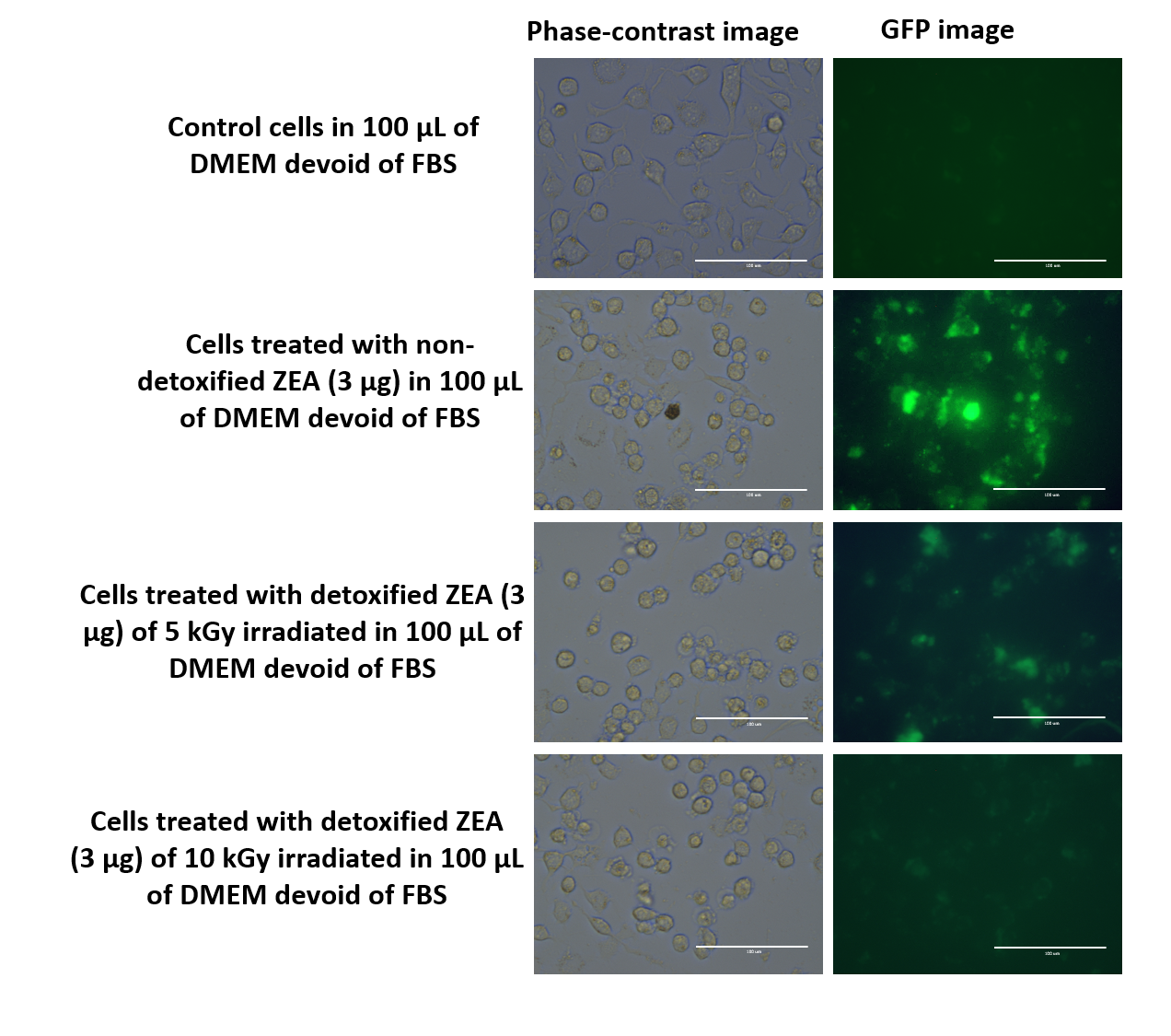

Supplement: Supplementary Figure S5 — Assessment of effect of non-detoxified and irradiation mediated detoxified zearalenone (ZEA) on generation of intracellular reactive oxygen species (ROS) in RAW 264.7 cells for 12 h by DCFH-DA staining. [file Image_5.TIF]

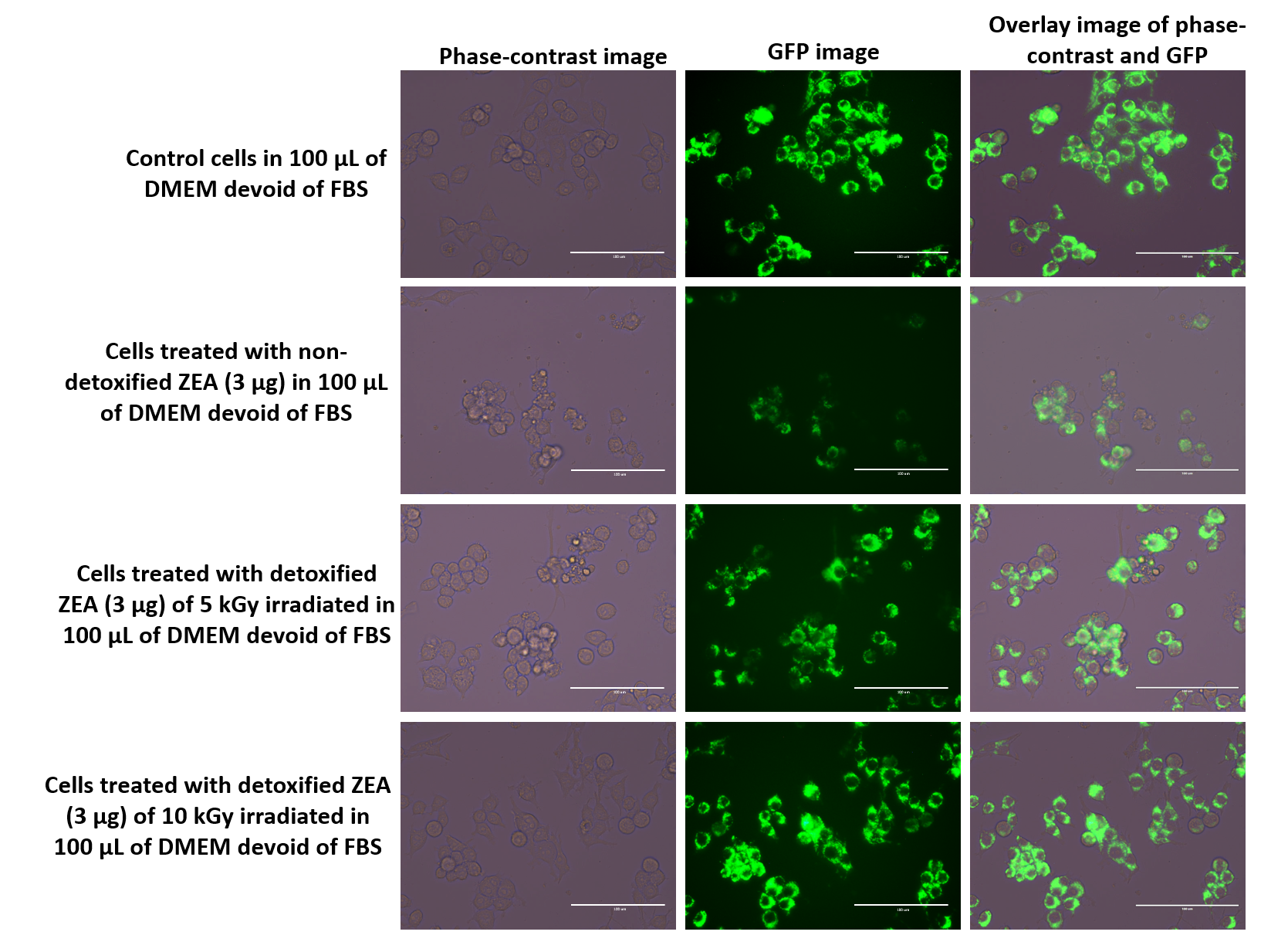

Supplement: Supplementary Figure S6 — Assessment of effect of non-detoxified and irradiation mediated detoxified zearalenone (ZEA) on mitochondrial membrane potential (MMP) in RAW 264.7 cells for 12 h by rhodamine 123 staining. [file Image_6.TIF]

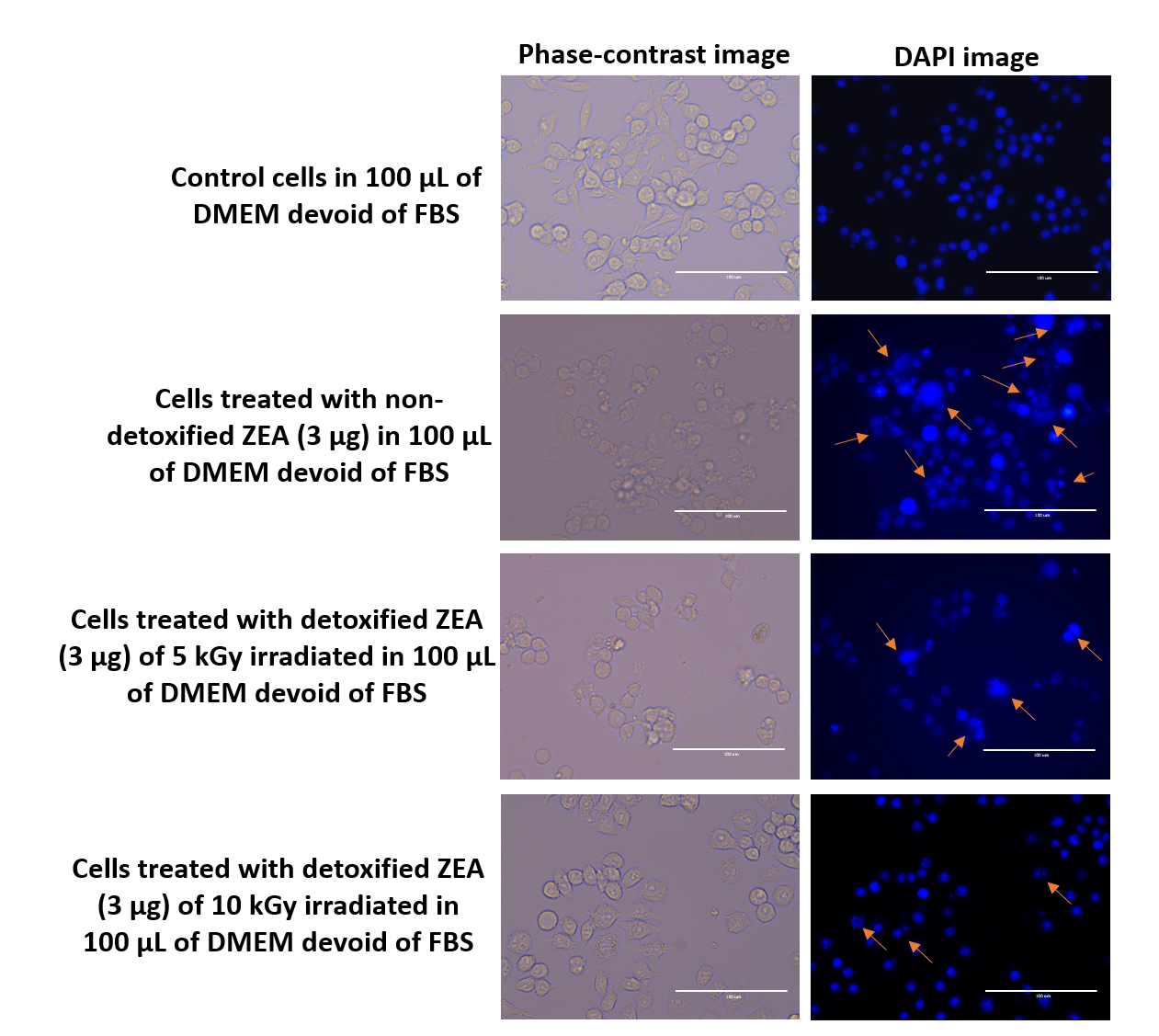

Supplement: Supplementary Figure S7 — Assessment of effect of non-detoxified and irradiation mediated detoxified zearalenone (ZEA) on nuclear damage in RAW 264.7 cells for 12 h by DAPI staining. [file Image_7.TIF]
